# *Arabidopsis* Homologs of Retinoblastoma-Associated Protein 46/48 Associate with a Histone Deacetylase to Act Redundantly in Chromatin Silencing

**DOI:** 10.1371/journal.pgen.1002366

**Published:** 2011-11-10

**Authors:** Xiaofeng Gu, Danhua Jiang, Wannian Yang, Yannick Jacob, Scott D. Michaels, Yuehui He

**Affiliations:** 1 Department of Biological Sciences, National University of Singapore, Singapore, Singapore; 2 Temasek Life Sciences Laboratory, Singapore, Singapore; 3 Department of Biology, Indiana University, Bloomington, Indiana, United States of America; University of California Riverside, United States of America

## Abstract

RNA molecules such as small-interfering RNAs (siRNAs) and antisense RNAs (asRNAs) trigger chromatin silencing of target loci. In the model plant *Arabidopsis*, RNA–triggered chromatin silencing involves repressive histone modifications such as histone deacetylation, histone H3 lysine-9 methylation, and H3 lysine-27 monomethylation. Here, we report that two *Arabidopsis* homologs of the human histone-binding proteins Retinoblastoma-Associated Protein 46/48 (RbAp46/48), known as MSI4 (or FVE) and MSI5, function in partial redundancy in chromatin silencing of various loci targeted by siRNAs or asRNAs. We show that *MSI5* acts in partial redundancy with *FVE* to silence *FLOWERING LOCUS C* (*FLC*), which is a crucial floral repressor subject to asRNA–mediated silencing, *FLC* homologs, and other loci including transposable and repetitive elements which are targets of siRNA–directed DNA Methylation (RdDM). Both FVE and MSI5 associate with HISTONE DEACETYLASE 6 (HDA6) to form complexes and directly interact with the target loci, leading to histone deacetylation and transcriptional silencing. In addition, these two genes function in de novo CHH (H = A, T, or C) methylation and maintenance of symmetric cytosine methylation (mainly CHG methylation) at endogenous RdDM target loci, and they are also required for establishment of cytosine methylation in the previously unmethylated sequences directed by the RdDM pathway. This reveals an important functional divergence of the plant RbAp46/48 relatives from animal counterparts.

## Author Summary

Chromatin, made of histones and DNA, is often covalently modified in the nucleus, and modifications can regulate gene transcription. RNA molecules such as small-interfering or silencing RNAs (siRNAs) and antisense RNAs (asRNAs) can trigger silencing of gene expression in eukaryotes. We have found that in the flowering plant *Arabidopsis*, two homologous putative histone-binding proteins associate with a histone deacetylase and function in partial redundancy in chromatin-based silencing of various loci targeted by siRNAs or asRNAs. They act in partial redundancy to silence a development-regulatory gene that controls the transition to flowering and whose silencing is triggered by asRNAs, and genomic loci containing transposable and repetitive elements whose silencing is triggered by siRNAs via the siRNA–directed DNA Methylation (RdDM) pathway. In addition, these two genes function in maintenance of DNA methylation at RdDM loci and are also required for establishment of DNA methylation in the previously unmethylated sequences, revealing that histone modifications are partly required for DNA methylation. Our findings implicate that RNA–triggered transcriptional silencing involves repressive histone modifications such as deacetylation at a target locus.

## Introduction

Cytosine DNA methylation is critical for stable silencing of transposable elements (*TE*) and repetitive sequences and for epigenetic regulation of endogenous gene expression in eukaryotes [1]–[3]. DNA methylation is thought to play an ancestral role in the defense against invasive DNA elements to maintain genome stability and integrity [1]–[3]. In the model plant *Arabidopsis*, cytosine methylation occurs in three different sequence contexts: CG, CHG and CHH. CG and CHG methylation are heritably maintained respectively by DNA METHYLTRANSFERASE 1 (MET1) and the plant-specific CHROMOMETHYLASE 3 (CMT3). CHH methylation is dynamically maintained through de novo methylation by the DOMAINS-REARRANGED METHYLTRANSFERASE 2 (DRM2) and the RdDM pathway [1].

RdDM is a mechanism by which siRNAs direct de novo cytosine methylation in all sequence contexts of target DNA sequences (complementary to the siRNAs). In *Arabidopsis*, the plant-specific RNA polymerase Pol IV is thought to initiate silencing by generating single-stranded RNA transcripts that are subsequently converted to double-stranded RNAs (dsRNAs) by RNA-DEPENDENT RNA POLYMERASE 2 (RDR2). dsRNAs are processed by DICER 3 (DCL3) to produce 24-nt siRNAs, which are subsequently loaded to an ARGONAUTE 4 (AGO4)-containing effector complex known as RISC (for RNA-Induced Silencing Complex). Through their interaction with long non-coding RNA transcripts from target loci, generated by the RNA polymerase Pol V, the loaded RISC complexes in association with DRM2 are targeted to RdDM target loci to establish cytosine methylation in CG, CHG and CHH contexts, leading to heterochromatin formation and transcriptional silencing [for reviews, see [2], [4].

siRNAs not only direct DNA methylation, but also trigger repressive histone modifications at RdDM target loci, including histone deacetylation, H3K9 dimethylation (H3K9me2) and H3K27 monomethylation (H3K27me1). Functional loss of the RISC component AGO4 causes a strong reduction in H3K9me2 at the endogenous RdDM target loci including transposable and repetitive elements [5], [6]. Furthermore, it has been shown that at RdDM target loci H3K27 monomethylation, a hallmark for silenced heterochromatin [7], is reduced upon loss of Pol V or AGO4 activity [5]. Together with DNA methylation, these repressive histone modifications establish a silenced heterochromatin state at RdDM target loci.

Histone modifications are involved in the control of DNA methylation. For instance, the H3K9 methyltransferase KRYPTONITE (KYP)/SUVH4 and SUVH4 homologs including SUVH2, SUVH5, SUVH6 and SUVH9, catalyze dimethylation of H3K9, which is recognized and bound by CMT3, leading to the maintenance of CHG methylation [1], [8]. Histone H3 lysine-4 (H3K4) demethylation is also involved in DNA methylation. Recent studies reveal that cytosine methylation is depleted in genomic regions with di- or tri-methylated H3K4 at a genome-wide level [9]; the H3K4 demethylase known as JMJ14/PKDM7B is required for H3K4 demethylation and CHG and CHH methylation at various RdDM target loci [10]. The histone deacetylase HDA6 deacetylates lysines of core histones including H3 and H4, and is required for cytosine methylation in transgenes and silenced *rRNA* genes [11]–[13]. Multiple genetic screens have revealed that *HDA6* is critical for transgene silencing [13], [14]. Loss of HDA6 activity causes a substantial decrease of symmetric cytosine methylation and a moderate reduction in asymmetric CHH methylation in an RdDM-silenced transgene promoter, leading to the transgene reactivation [13]. In addition, disruption of *HDA6* function gives rise to histone hyperacetylation and decreased CG and CHG methylation at silenced *rRNA* gene promoters [11]. HDA6 plays a dual role in silencing of these loci: deacetylating core histones and mediating cytosine methylation [15]. In this way, HDA6 and DNA methylation machinery are thought to work collaboratively to silence target loci.

The histone-binding proteins RbAp46 and RbAp48 are highly homologous WD40-repeat proteins and were first identified in mammalian cells as the tumor-suppressor Rb-binding proteins [16]. Subsequent studies revealed that RbAp46/48 is an integral subunit of multiple chromatin-modifying or -assembly complexes [for a review, see [16]]. RbAp46 forms a complex with the histone acetyltransferase called HAT1 that acetylates H4, whereas RbAp48 is a subunit of the Chromatin Assembly Factor-1 (CAF-1) complex that deposits nucleosomes. Both RbAp46 and RbAp48 are components of several histone deacetylase (HDAC) co-repressor complexes such as the Sin3 complex, which deacetylate core histones to repress target gene expression. In addition, RbAp46/48 is an integral subunit of the evolutionarily conserved Polycomb Repressive Complex 2 (PRC2)-like complexes that catalyze H3K27 trimethylation (H3K27me3), resulting in transcriptional repression. Recent studies have shown that RbAp46/48 functions as a histone (H3–H4 dimer)-binding protein [17]. It is believed that the RbAp46/48-containing complexes interact with histone substrates via either RbAp46 or RbAp48 [17].

RbAp46 and RbAp48 are evolutionarily conserved in animals and plants. There are five homologs in *Arabidopsis* known as MSI1–MSI5 (MSI for MULTICOPY SUPPRESSOR OF *IRA1*) [18]. Biological functions of MSI1 and MSI4/FVE have been identified, whereas the functions of MSI2, MSI3 and MSI5 are not known. *MSI1* is required for proper vegetative development and plays an essential role in gametophyte and seed development [19], [20]. The MSI1 protein is an integral subunit of the conserved *Arabidopsis* CAF-1 complex, and has also been found in several PRC2-like complexes [18], [20], [21]. In addition, MSI1 directly interacts with the *Arabidopsis* Rb homolog (RBR), a key cell-cycle regulator [22], to repress *MET1* expression in female gametogenesis, presumably resulting in a reduction in CG methylation [23]. In addition to MSI1, MSI4/FVE also interacts with a plant Rb homolog [24], but the biological implication of this interaction is unclear. *FVE* has been shown to repress expression of the central floral repressor *FLC* and several cold-responsive genes in *Arabidopsis* [24], [25]. *FLC* inhibits the transition from a vegetative to a reproductive phase (i.e. flowering), and loss of *FVE* function causes *FLC* de-repression, resulting in late-flowering [24], [25]. Previous studies reveal that *fve* mutations cause increased levels of histone acetylation at *FLC* chromatin [24], [26], indicating that *FVE* may be involved in deacetylation of *FLC* chromatin to repress *FLC* expression. However, recent studies show that loss of *FVE* function also gives rise to a strong reduction in PRC2-catalyzed H3K27me3, a repressive chromatin mark, in *FLC* chromatin [27]. Given that the human FVE homologs, RbAp46/48, are subunits of multiple histone-modifying complexes, the mechanisms underlying *FVE*-mediated transcriptional repression/silencing remain elusive.

*FLC* plays a crucial role in flowering-time regulation in *Arabidopsis* and *FLC* expression is affected by a range of chromatin modifiers (reviewed in refs 28,29). In most rapid-cycling (i.e. early flowering) *Arabidopsis* ecotypes, *FLC* expression is repressed or silenced by a group of proteins that mediate or trigger repressive histone modifications at the *FLC* locus, among which, in addition to FVE, are two conserved RNA 3′end-processing factors called CstF64 and CstF77, RNA-binding proteins known as FCA and FPA, a putative H3K4 demethylase FLOWERING LOCUS D (FLD), and a putative CLF (for CURLY LEAF)-containing PRC2-like complex [for reviews, see [28], [29]]. Furthermore, recent studies show that *FLC* antisense transcripts trigger *FLC* silencing [30], [31]. There are two groups of antisense transcripts resulting from alternative polyadenylation. *CstF64* and *CstF77* function together with *FCA* and *FPA* to promote polyadenylation of *FLC* antisense transcripts at a proximal site, triggering *FLC* silencing [30], [31]. FLD activity is required for, and acts downstream *FCA* and *FPA* in this silencing mechanism [30], [32]. It is believed that the 3′ processing at the proximal polyadenylation site on *FLC* antisense transcripts leads to co-transcriptional decay of the antisense RNA downstream the proximal site, which may generate aberrant RNAs and trigger repressive histone modifications such as *FLD*-mediated H3K4 demethylation, and consequent silencing [30].

*FLC* antisense transcript-triggered silencing is mechanistically different from the siRNA-triggered silencing of RdDM target loci, although both involve RNA molecules. So far, no siRNAs targeting the *FLC* genomic coding region or 5′ promoter have been detected in *Arabidopsis*. Consistent with this, knockout of siRNA-silencing pathway components such as *Pol IV*, *Pol V*, *RDR2* or *AGO4* has little effect on *FLC* silencing [33]. In addition, cytosines in genomic *FLC* in most *Arabidopsis* ecotypes are not methylated [34]. Thus, unlike RdDM-mediated silencing, cytosine methylation is not directly involved in *FLC* silencing. However, both silencing mechanisms require repressive histone modifications such as histone deacetylation, H3K4 demethylation, and/or H3K9 and H3K27 methylation, and involve chromatin silencing.

In this study, we explored the role for FVE and MSI5 in chromatin silencing of various loci targeted by siRNAs or asRNAs. We show that *MSI5* acts in partial redundancy with *FVE* to silence *FLC* and endogenous RdDM target loci including *FWA* (containing two tandem repeats), *AtMu1* (DNA transposon), *AtSN1* (retrotransposon) and *IG/LINE* (intergenic transcripts). FVE and MSI5 associate with the histone deacetylase HDA6 to form HDAC complexes, and directly interact with the target loci, leading to histone deacetylation and transcriptional silencing. Together, these results show that *FVE* and *MSI5* play an important role in the chromatin silencing of various loci targeted by siRNAs or asRNAs in plants.

## Results

### *MSI5* Functions Redundantly with *FVE* to Promote the Floral Transition

FVE and MSI5 are *Arabidopsis* homologs of the human histone-binding RbAp46/48 [18], [24]. The amino acid sequence similarity between FVE and RbAp48 over the entire RbAp48 is 45%, and the similarity between MSI5 and RbAp48 over the entire RbAp48 is also 45%, whereas the identity between FVE and MSI5 over the entire MSI5 is 77% (Figure S1). The high degree of sequence conservation between MSI5 and FVE suggests that these two proteins may have a similar biochemical function.

**Figure 1:**
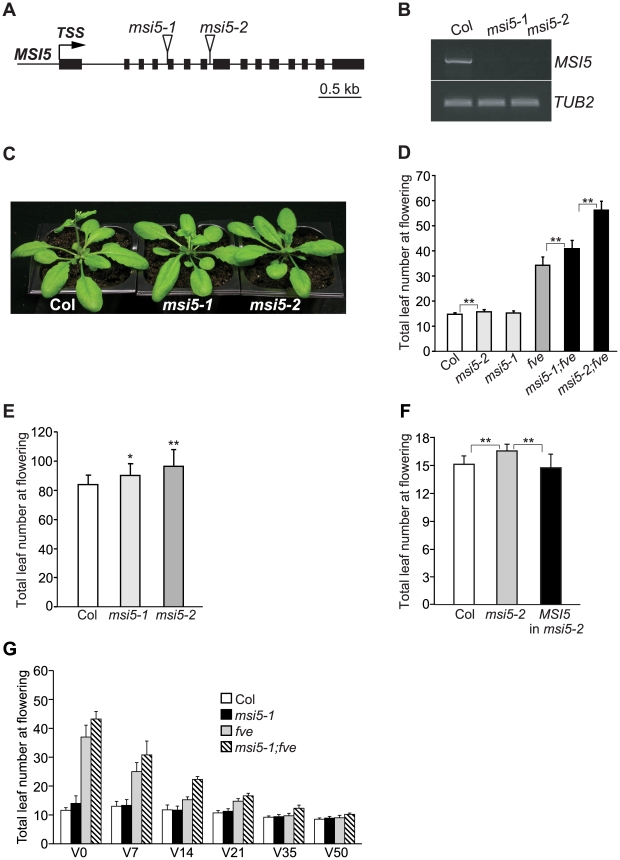
Characterization of *msi5* Mutants. (A) *MSI5* gene structure. Exons are represented by filled boxes, and the transcription start site (TSS) is indicated with an arrow. Triangles indicate *T-DNA* insertion sites. (B) RT-PCR analysis of the full-length *MSI5* transcript levels in *msi5* mutant seedlings. *TUBLIN2* (*TUB2*) serves as the endogenous control. (C) *msi5* mutants grown in LD. (D) Flowering times of the indicated genotypes grown in LD. Double asterisks indicate statistically significant differences in the means between indicated genotypes as revealed by two-tailed Student's t-test (**, P<0.01). (E) Flowering times of *msi5* mutants grown in short days. Asterisks indicate statistically significant differences in the means between *msi5* mutants and Col (*, P<0.05; **, P<0.01). (F) Complementation of the *msi5-2* allele by the wild-type *MSI5* gene. Flowering times of Col, *msi5-2* and *msi5-2* carrying the wild-type *MSI5* (T_1_ generation) grown in LD, were scored. Asterisks indicate statistically significant differences in the means between indicated genotypes. (G) Effects of cold treatments on the flowering times of the indicated genotypes grown in LD. Seedlings were treated at 4°C; “V” indicates days of cold treatment before the plants returned to normal growth condition. (D–G) Flowering times are expressed as the total number of primary rosette and cauline leaves at flowering; for each genotype, at least 10 plants were scored. Error bars indicate standard deviation (SD).

Previous studies have shown that *FVE* represses the floral transition in *Arabidopsis* [24], [25]. We sought to address the biological functions of *MSI5*. Two loss-of-function mutants of *MSI5* carrying insertional *T-DNA*s were identified, in which the full-length transcription of *MSI5* was severely disrupted (Figure 1A and 1B). Grown in long days (LD; 16-hr light/8-hr dark), *msi5-1* did not exhibit any visible phenotypes, whereas *msi5-2* flowered slightly later than wild-type Col (Figure 1C and 1D), as measured by the developmental criterion of the number of leaves formed prior to flowering, from the primary apical meristem. In short days (8-hr light/16-hr dark), both mutants flowered moderately later than Col (Figure 1E). In both long and short days, *msi5-2* flowered later than *msi5-1*, indicating that *msi5-2* is a strong allele. We further confirmed that the moderate late-flowering of *msi5-2* was indeed caused by the mutation in a complementation test in which the wild-type copy of *MSI5* complemented the *msi5-2* mutation (Figure 1F). To examine whether *MSI5* acts redundantly with *FVE* to repress flowering, we introduced both *msi5* alleles into *fve* mutants. In LDs, both *msi5-1;fve* and *msi5-2;fve* flowered later than the late-flowering *fve* mutants (Figure 1D). Of note, *msi5-2;fve* flowered with 56 leaves on average which is much later than *fve* (34 leaves on average) (Figure 1D). Hence, *MSI5* functions redundantly with *FVE* to promote *Arabidopsis* flowering.

Vernalization (an extended period of cold exposure) promotes *Arabidopsis* flowering. We examined the effect of cold treatment on the flowering times of *msi5-1;fve*. The late flowering phenotypes of this double mutant were partially suppressed by 7-day cold treatment, and after 35 days of cold exposure, the mutant flowered similar to Col (Figure 1G). It is well known that vernalization largely represses *FLC* expression to accelerate flowering in *Arabidopsis* [28], [29]. These data indicate that the late-flowering phenotypes of *msi5;fve* is largely dependent on *FLC* and that the activities of *MSI5* and *FVE* are not required for *FLC* repression by vernalization.

### *MSI5* Functions in Partial Redundancy with *FVE* to Repress the Expression of *FLC* and *FLC* Homologs

**Figure 2:**
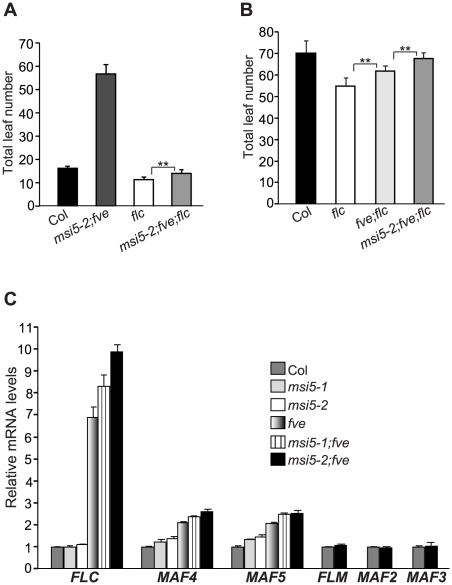
*MSI5* and *FVE* Act in Partial Redundancy to Repress the Expression of *FLC* and *FLC* Homologs. (A–B) Flowering times of the indicated genotypes grown in LD (A) and short days (B). 14–21 plants grown in LD and 6–11 plants grown in short days, were scored for each genotype. Error bars indicate SD. Double asterisks indicate statistically significant differences in the means between indicated genotypes as revealed by two-tailed Student's t-test (**, P<0.01). (C) Relative mRNA levels of *FLC* and *FLC* homologs in the seedlings of indicated genotypes as quantified by real-time PCR. The transcript levels of each gene were normalized to the endogenous control *TUB2*. Relative expression to wild-type Col is presented. Bars indicate SD.

*FVE* has been shown to repress *FLC* expression [24], [25]. To examine whether the late flowering of *msi5;fve* was caused by *FLC* de-repression, we created an *flc;msi5-2;fve* triple mutant. In long days, the triple mutant flowered much earlier than *msi5-2;fve*, but still moderately later than *flc* (Figure 2A). Hence, the late-flowering of *fve;msi5-2* is partly dependent on *FLC*. We further examined the flowering times of *flc*, *fve;flc* and *flc;msi5-2;fve* in short days, and found that the triple mutant flowered later than *fve;flc* and *flc* (Figure 2B), suggesting that *FVE* and *MSI5* may repress other floral repressors to promote flowering, in addition to *FLC*.

Besides *FLC*, *Arabidopsis* has five *FLC* homologs including *FLOWERING LOCUS M* (*FLM*) (also known as *MAF1*), and *MAF2-MAF5* (*MAF* for *MADS BOX AFFECTING FLOWERING*); these genes moderately repress flowering [35]–[37]. We quantified transcript levels of *FLC* and *FLC* homologs in Col, *msi5*, *fve*, and *msi5;fve* seedlings. *FLC* expression was slightly increased in *msi5-2* compared to Col, whereas it remained unchanged in *msi5-1* (Figure 2C). However, both *msi5* alleles caused strong increases in *FLC* transcript levels in the *fve* background (Figure 2C). Furthermore, we found that in *fve* mutants both *MAF4* and *MAF5* were de-repressed, and this de-repression was enhanced upon loss of *MSI5* function in the *fve* background, whereas *MAF1*, *MAF2* and *MAF3* expression remained unchanged upon loss of *FVE* and *MSI5* function (Figure 2C). Together, these data show that *MSI5* acts redundantly with *FVE* to repress the expression of *MAF4* and *MAF5*, in addition to *FLC*, and promote the floral transition.

### *MSI5* Acts Redundantly with *FVE* to Silence Endogenous RdDM Target Loci

**Figure 3:**
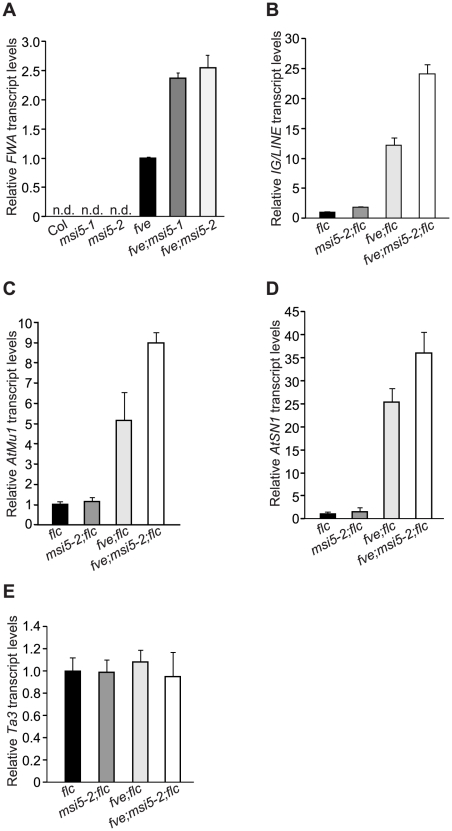
*MSI5* Acts Redundantly with *FVE* to Silence Endogenous RdDM Target Loci. (A) Relative *FWA* mRNA levels in the seedlings of indicated genotypes, quantified by real-time RT-PCR. Relative expression to the *fve* mutant is presented. “n.d.” indicates that *FWA* transcript levels were too low to be detected. The transcript levels were normalized to the endogenous control *TUB2*. Bars indicate SD. (B–E) Relative transcript levels of *IG/LINE* (B), *AtMu1* (C), *AtSN1* (D) and *Ta3* (E) in the seedlings of indicated genotypes as quantified by real-time PCR. Relative expression to the *flc* mutant is presented. Bars indicate SD.

Recent genetic analyses have revealed that *FVE* is partly required for proper silencing of the RdDM target loci *AtSN1* (retrotransposon) and *AtMu1* (DNA transposon), although the underlying mechanism is unknown [38], [39]. This prompted us first to explore whether *FVE* plays a broad role in silencing of the RdDM target loci including *TE*s and repetitive elements. We examined the effect of loss of *FVE* function on the silencing of two other representative RdDM loci, *FWA* and *IG/LINE*. *FWA*, encoding a homeodomain-containing transcription factor that can repress flowering, is sporophytically silenced by cytosine methylation in two sets of tandem repeats containing a sequence related to a SINE (for Short Interspersed Nuclear Element) retroelement located in the 5′ region of *FWA* [40], [41]. *IG/LINE* is a spurious intergenic transcript initiated from a flanking *solo-LTR* (for Long Terminal Repeat) that functions as a promoter [42]. Upon loss of *FVE* function, *FWA* and *IG/LINE* were re-activated in the *fve* or *fve;flc* seedlings, respectively (Figure 3A, 3B). We asked whether *MSI5* was required for silencing of RdDM target loci. To this end, we first quantified the transcript levels of *AtSN1*, *AtMu1* and *IG/LINE* in *msi5-2;flc* and *msi5-2;fve;flc* seedlings. Both *msi5-2* and *msi5-2;fve* were introduced into the *flc* background to exclude the possibility that *FLC* de-repression may affect reactivation of these loci. Loss of *MSI5* function alone had little effect on silencing of these three loci; however, upon the combined loss of *FVE* and *MSI5* function, all three loci were strongly re-activated to levels much higher than *fve* alone (Figure 3B–3D). Next, we measured *FWA* transcript levels in *msi5* and *msi5;fve* seedlings [in the Col background; note that *FLC* upregulation does not affect *FWA* silencing [43]]. *FWA* is fully silenced in the *msi5* seedlings (Figure 3A), but the *msi5* mutations greatly enhanced *FWA* reactivation upon loss of *FVE* function (Figure 3A), like the situation in the other three loci. Together, these data suggest that *MSI5* and *FVE* may play a broad role in silencing of transposable and repetitive elements in *Arabidopsis* genome, and that *MSI5* functions redundantly with *FVE* to silence these elements.

These distinct four loci have a common feature, that is, their de novo silencing is established by the siRNA-triggered DNA methylation pathway [40], [42], [44], [45]. To test whether *MSI5* and *FVE* were involved in silencing of *TE*s other than RdDM target loci, we examined the transcript levels of *Ta3* in *msi5* and/or *fve* mutant seedlings (in the *flc* background), which is a pericentromeric *TE* that is silenced independently of siRNAs [46]. Loss of *MSI5* and/or *FVE* function did not cause *Ta3* reactivation (Figure 3E). These data indicate that *MSI5* and *FVE* may only be required for the silencing of RdDM-targeted *TE*s and repetitive elements.

### *MSI5* and *FVE* Are Required for Both De Novo Asymmetric Cytosine Methylation and Maintenance of Symmetric Methylation at Endogenous RdDM Loci

**Figure 4:**
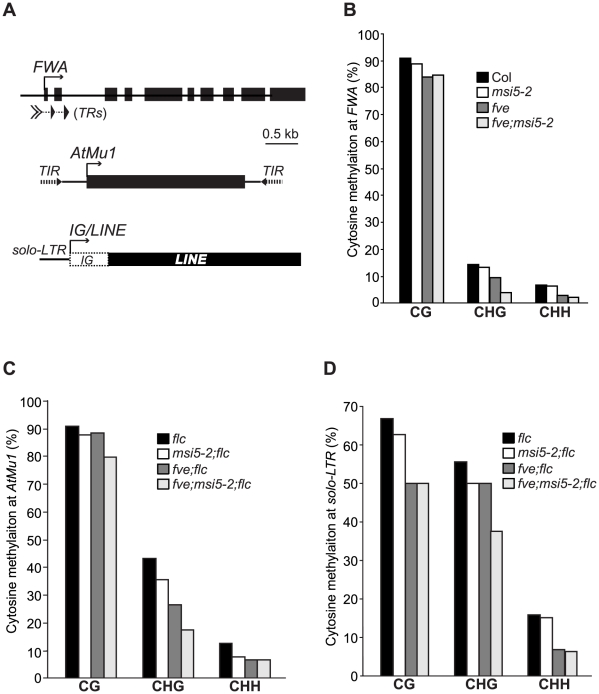
Analysis of DNA Methylation at Endogenous RdDM Target Loci. (A) Schematic structures of genomic *FWA*, *AtMu1* and *IG/LINE*. Boxes represent exons at *FWA* and *AtMu1* or transcribed regions at *IG/LINE*. A solid arrow indicates TSS. (B) Cytosine methylation at *FWA* tandem repeats (*TR*s) as determined by bisulfite sequencing. (C) Cytosine methylation at *AtMu1 TIR* (3′). (D) Cytosine methylation at *solo-LTR*. (B–D) For each genotype, 8–17 plasmids derived from bisulfite-converted genomic DNA were sequenced and examined.

*FWA*, *AtMu1* and *IG/LINE* are silenced by cytosine methylation [40]–[42], [44]. We sought to determine whether *MSI5* and *FVE* are required for cytosine methylation in these loci. Using bisulfite sequencing, we examined cytosine methylation at the tandem repeats (*TR*s), terminal inverted repeats (*TIR*s) and *solo-LTR*, respectively, in *FWA*, *AtMu1* and *IG/LINE* in *msi5* and/or *fve* mutant seedlings (note that these repeats generate siRNAs) (Figure 4A). At the *FWA* locus, CG methylation was slightly reduced, but a strong reduction in CHG and CHH methylation was observed, in *msi5-2;fve* compared to wildtype (^m^CHG: 14% in WT, but 4% in *msi5-2;fve*; ^m^CHH: 7% in WT, but 2% in *msi5-2;fve*); neither CHG nor CHH methylation was affected in *msi5-2*, whereas upon loss of *FVE* function *CHG* and *CHH* methylation was moderately reduced (Figure 4B). At *AtMu1*, CG methylation was not affected, but CHG methylation was greatly reduced in *msi5-2;fve* (in the *flc* background); in addition, CHH methylation was moderately reduced upon loss of *FVE* and *MSI5* function (Figure 4C). At *solo-LTR*, cytosine methylation in all contexts was reduced upon the combined loss of *FVE* and *MSI5* function (Figure 4D). The reduction of non-CG methylation at the *FWA*, *AtMu1* and *solo-LTR* loci was further confirmed using the methylation-sensitive restrictive endonucleases *Fnu4HI* or *AluI* (Figure S2). Together, these results show that *MSI5* and *FVE* primarily mediate CHH and CHG methylation at RdDM target loci.

Recent studies show that symmetric CHG methylation is largely maintained by CMT3 in concert with the H3K9 methyltransferase KYP, whereas CHH methylation cannot be maintained, but is de novo methylated by the RdDM pathway [1]. Hence, we conclude that *MSI5* and *FVE* are required for the de novo CHH methylation and maintenance of CHG methylation at the RdDM target loci. Cytosine methylation at both *AtMu1 TIR*s and *solo-LTR* causes transcriptional silencing. The reduction in cytosine methylation at the non-transcribed and siRNA-targeted regions (*TIR*s and *solo-LTR*) upon loss of *FVE* and *MSI5* function, suggests that these two genes silence RdDM target loci partly by mediating DNA methylation in these loci.

### *FVE* and *MSI5* Contribute to the Establishment of DNA Methylation of the *FWA* Transgene Newly Introduced into *Arabidopsis* Genome

Both *FVE* and *MSI5* are required for de novo CHH methylation at the endogenous RdDM target loci. We sought to examine whether they could be involved in de novo cytosine methylation in all sequence contexts on previously unmethylated sequences using an *FWA* transgene assay. When an unmethylated *FWA* transgene is introduced into *Arabidopsis* genome, siRNAs from the endogenous *FWA* are able to target the transgene and through the RdDM pathway direct de novo cytosine methylation in all sequence contexts, leading to its silencing [40], [47]. Otherwise, ectopic *FWA* expression would give rise to a late-flowering phenotype [47], [48].

**Figure 5:**
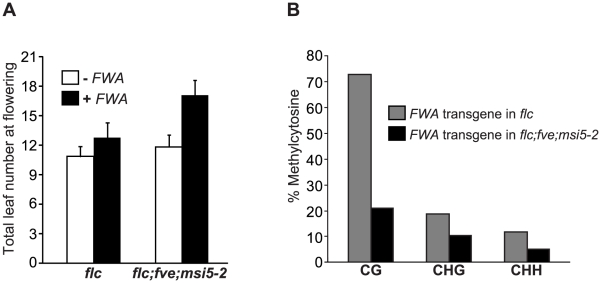
*MSI5* and *FVE* Are Required for De Novo Silencing of the *FWA* Transgene. (A) Flowering times of *flc* and *flc;msi5-2;fve* mutants transformed with an *FWA* transgene. Total leaf number for each line or T_1_ transgenic population with 15–16 plants grown in LD, were scored. Bars indicate SD. (B) Cytosine methylation at the tandem repeats of *FWA* transgene (in T_1_ transformants) determined by bisulfite sequencing. For each genotype, 15 plasmids derived from bisulfite-converted genomic DNA were sequenced and examined.

We introduced an *FWA* transgene [47], [48] into *flc* and *flc;msi5-2;fve* mutant backgrounds. Consistent with our previous finding that *FWA* transgene is de novo silenced in the *flc* background [43], T_1_ transformants of the *flc* background flowered only slightly later than *flc* (Figure 5A). By contrast, T_1_ transformants of the *flc;msi5-2;fve* mutant flowered much later than the non-transformed control (Figure 5A). Hence, *MSI5* and *FVE* are required for de-novo silencing of the incoming *FWA* transgene.

We further examined the methylation state of *FWA* transgene in the *flc* and *flc;msi5-2;fve* backgrounds by bisulphite sequencing, and observed that CG methylation (a primary contributor for *FWA* silencing), was significantly reduced in the transgene upon the combined loss of *FVE* and *MSI5* function (Figure 5B), in contrast to the slight reduction in CG methylation of the endogenous *FWA* (Figure 4B). In addition, non-CG methylation of *FWA* transgene was also reduced in *flc;msi5-2;fve* compared to the *flc* background. Together, these results show that *FVE* and *MSI5* are required for the establishment of cytosine methylation in all sequence contexts of the newly introduced *FWA* transgene and thus de novo *FWA* silencing.

### *MSI5* Spatial Expression Pattern Overlaps That of *FVE*

**Figure 6:**
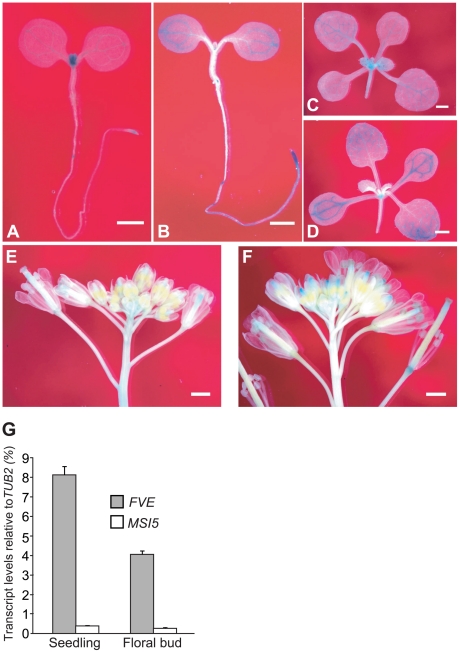
Expression Patterns of *FVE* and *MSI5*. (A–B) Spatial expression patterns of *MSI5-GUS* (A) and *FVE-GUS* (B) in 4-d-old seedlings. Seedlings were stained in a staining buffer with 0.5-mg 5-bromo-4-chloro-3-indolyl-β-d-glucuronic acid (X-Gluc) at 37°C for 6 hr. (C–D) Spatial expression patterns of *MSI5-GUS* (C) and *FVE-GUS* (D) in 10-d-old seedlings (stained for 6 hr). (E–F) Spatial expression patterns of *MSI5-GUS* (E) and *FVE-GUS* (F) in inflorescence (stained for 8 hr). (A–F) Bars indicate 1.0 mm. (G) Relative mRNA levels of *FVE* and *MSI5* in the wildtype (Col) seedlings and floral buds quantified by real-time PCR. The transcript levels of each gene were normalized to the endogenous control *TUB2*.

The functional redundancy of *MSI5* with *FVE* raised the possibility that both genes could be expressed in the same tissues. To test this, we examined the spatial expression patterns of *MSI5* and *FVE* using translational fusions to the reporter gene *β-GLUCURONIDASE* (*GUS*); the constructs contained the promoter plus part of the protein-coding region of *FVE* or *MSI5*. In seedlings, both *MSI5* and *FVE* were preferentially expressed in shoot apices, root tips and leaf vasculature (Figure 6A–6D). In the reproductive phase, both genes were mainly expressed in styles and the junctions of ovary and receptacle (Figure 6E, 6F). In general, *FVE-GUS* was expressed at a level higher than that of *MSI5-GUS*. We confirmed that indeed, *FVE* transcript levels were much higher than those of *MSI5* in both seedlings and floral buds (Figure 6G). This may partly explain why *FVE* plays a more dominant role in gene silencing than *MSI5* does. Given the high protein-sequence homology of MSI5 with FVE, the overlapping expression patterns of these two genes provide an explanation for the functional redundancy of *MSI5* with *FVE*.

### Both FVE and MSI5 Associate with HDA6 to Form HDAC Complexes

**Figure 7:**
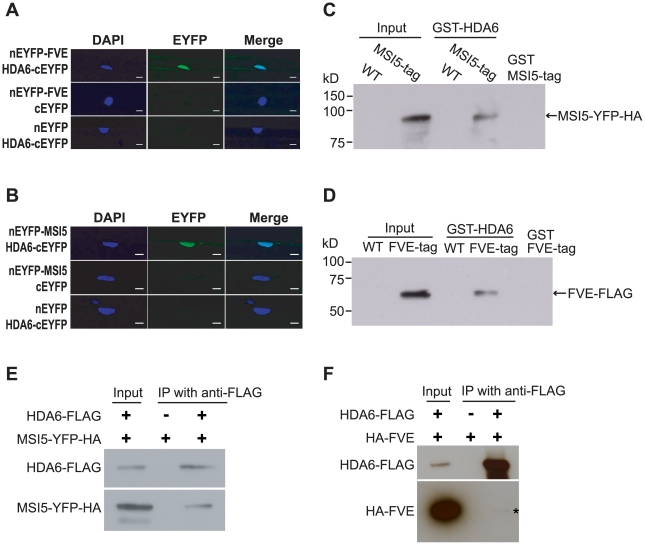
HDA6 Forms a Nuclear Complex with MSI5 or FVE. (A) BiFC analysis of nEYFP-FVE and HDA6-cEYFP in onion epidermal cells. Each pair of plasmids was used to transiently co-transform onion epidermal cells by biolistic gene bombardment. Yellowish-green signal indicates the binding of nEYFP-FVE with HDA6-cEYFP in the nuclei. Blue fluorescence from DAPI (4′,6-diamidino-2-phenylindole) staining indicates nuclei. Fluorescence signals were imaged using a laser scanning confocal microscope. Bar = 20 µm. (B) BiFC analysis of nEYFP-MSI5 and HDA6-cEYFP in onion epidermal cells. Bar = 20 µm. (C–D) GST-HDA6 pull-down assays. Total proteins were extracted from wildtype (Col) and transgenic seedlings expressing MSI5-YFP-HA or FVE-FLAG, and subsequently were incubated with the purified GST-HDA6. Proteins were recovered using glutathione-linked resins and analyzed by immunoblotting with anti-HA (recognizing MSI5-YFP-HA) and anti-FLAG (recognizing FVE-FLAG) antibodies. Note that GST-HDA6 does not directly interact with YFP-HA (data not shown). (E) Co-immunoprecipitation of HDA6 with MSI5 in seedlings. Total protein extracts from seedlings of the *MSI5-YFP-HA* line (a negative control) and F_1_ of the doubly hemizygous *MSI5-YFP-HA* and *HDA6-FLAG*, were immunoprecipitated with anti-FLAG agarose; subsequently, the precipitates were analyzed by western blotting with anti-FLAG (recognizing HDA6-FLAG) and anti-HA (recognizing MSI5-YFP-HA). The input was the protein extract of doubly hemizygous *MSI5-YFP-HA* and *HDA6-FLAG*. (F) Co-immunoprecipitation of HDA6 with FVE in seedlings. Total protein extracts from seedlings of the *HA-FVE* line (a negative control) and F_1_ of the doubly hemizygous *HA-FVE* and *HDA6-FLAG*, were immunoprecipitated with anti-FLAG agarose recognizing HDA6-FLAG. HA-FVE protein was specifically detected in the precipitates from the *HA-FVE* and *HDA6-FLAG*-expressing seedlings, but not from the seedlings expressing only *HA-FVE*.

The mammalian homologs of MSI5 and FVE, RbAp46/48, are subunits of several chromatin-modifying complexes involved in gene silencing such as HDAC co-repressor complexes; RbAp46/48 binds H3–H4 dimers and is thought to recognize and bind histone substrates in these complexes [17]. Using a candidate-gene approach, we explored whether FVE and/or MSI5 could associate with HDA6, an HDAC that has been shown to be involved in *FLC* repression, DNA methylation maintenance and gene silencing in *Arabidopsis* [11], [13], [49]. Bimolecular fluorescence complementation (BiFC) [50] was employed to examine whether MSI5 and FVE could associate with HDA6 in plant cells. A non-fluorescent N-terminal EYFP (for Enhanced Yellow Fluorescent Protein) fragment was fused to the full-length FVE and MSI5 individually, whereas a non-fluorescent C-terminal EYFP fragment was fused to the full-length HDA6. nEYFP-FVE and HDA6-cEYFP were simultaneously expressed in onion epidermal cells, and fluorescence was observed in the nuclei, reflecting the physical association of FVE with HDA6 in the nucleus (Figure 7A). Similarly, we also found that MSI5 associated with HDA6 in the nuclei of onion cells (Figure 7B).

Next, we performed protein pull-down assays to confirm the association of HDA6 with FVE and MSI5. Transgenic lines (T_3_ homozygotes) expressing *MSI5-YFP-HA* (in *msi5-2* background) or *FVE-FLAG* (in *fve* background) were created. The *MSI5* transgene was fully functional (Figure S3A), whereas the *FVE-FLAG* was partially functional (Figure S3B. Total proteins were extracted from transgenic seedlings and mixed with the purified GST-HDA6 from *E.coli*. HDA6 was able to pull down the MSI5 fusion and FVE-FLAG from the protein extracts (Figure 7C–7D). Thus, HDA6 can directly associate with FVE and MSI5 from *Arabidopsis* seedlings.

We further performed co-immunoprecipitation (co-IP) experiments to determine whether HDA6 is part of a complex with FVE or MSI5 *in vivo*. First, we created transgenic lines expressing a functional HDA6-FLAG (Figure S3C), and a line expressing a functional HA-FVE (Figure S3D). *HDA6-FLAG*-expressing plants were crossed to the *HA-FVE* line or the *MSI5-YFP-HA* line, and from the resulting F_1_ seedlings total proteins were extracted for co-IP analysis. Indeed, we found that anti-FLAG (recognizing HDA6-FLAG) immunoprecitated the MSI5 fusion protein and HA-FVE from the seedlings (Figure 7E, 7F). Of note, we detected only a small portion of the HA-FVE protein in the HDA6-FLAG immunoprecipitates from the F_1_ seedlings (note that no HA-FVE was immunoprecipitated from the seedlings expressing only HA-FVE); this is most likely due to an unstable association of FVE with HDA6. Taken together, these results led us to infer that FVE or MSI5 forms an HDAC complex with HDA6 in *Arabidopsis*.

### *HDA6* Is Required for DNA Methylation and Silencing of *FVE* and *MSI5* Target Loci

**Figure 8:**
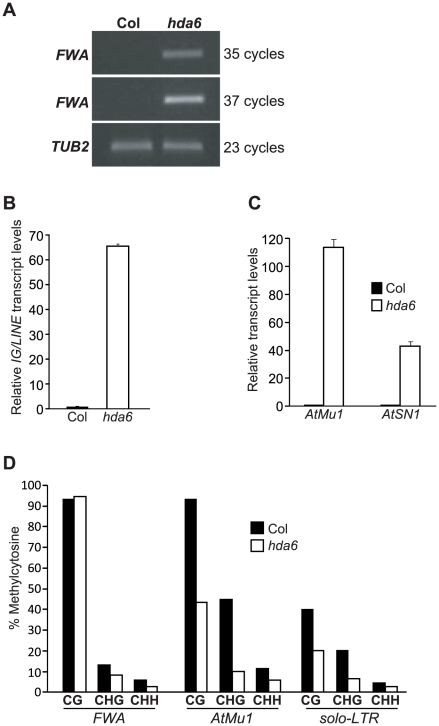
Analysis of Reactivation of Silent RdDM Target Loci in the *hda6* Mutant. (A) RT-PCR analysis of *FWA* expression in Col (WT) and *hda6* mutants. (B–C) Relative transcript levels of *IG/LINE*, *AtMu1* and *AtSN1* in Col and *hda6* seedlings as quantified by real-time RT-PCR. The transcript levels were normalized to the endogenous control *TUB2*. Relative expression to Col is presented. Bars indicate SD. (D) Cytosine methylation at *FWA* tandem repeats, *AtMu1 TIR* (3′) and *solo-LTR* in the *hda6* mutant, as determined by bisulfite sequencing. 15 plasmids derived from bisulfite-converted genomic DNAs were sequenced and examined.

Consistent with the HDA6 association with FVE and MSI5, recent studies show that *HDA6*, like *FVE* and *MSI5*, represses *FLC*, *MAF4* and *MAF5* expression to promote the floral transition [49]. It was of interest therefore to determine whether *HDA6* also silences endogenous RdDM target loci. We measured transcript levels of *FWA*, *AtMu1*, *AtSN1* and *IG/LINE* in WT (Col) and *hda6* seedlings. Indeed, loss of HDA6 activity, like loss of *MSI5* and *FVE* function, caused re-activation of all four loci (Figure 8A–8C). Thus, *HDA6*, like *MSI5* and *FVE*, is required for silencing of the RdDM target loci.

We further examined cytosine methylation state in the *FWA*, *AtMu1* and *solo-LTR* loci in *hda6* seedlings. At *FWA*, loss of *HDA6* function, like of loss of *FVE* and *MSI5* function, caused a reduction in CHG and CHH methylation (Figure 8D). At *solo-LTR*, cytosine methylation in all sequence contexts was reduced in *hda6* compared to wildtype (Figure 8D), similar to the situation in the *msi5-2;fve* mutant (Figure 4D). In addition, at *AtMu1*, upon loss of *HDA6* function cytosine methylation in all sequence contexts was reduced (Figure 8D). Together, these results show that *HDA6*, like *MSI5* and *FVE*, is required for cytosine methylation at these RdDM target loci.

### FVE Protein Directly Interacts with *FLC* and RdDM Loci and Is Required for Histone Deacetylation at These Loci

**Figure 9:**
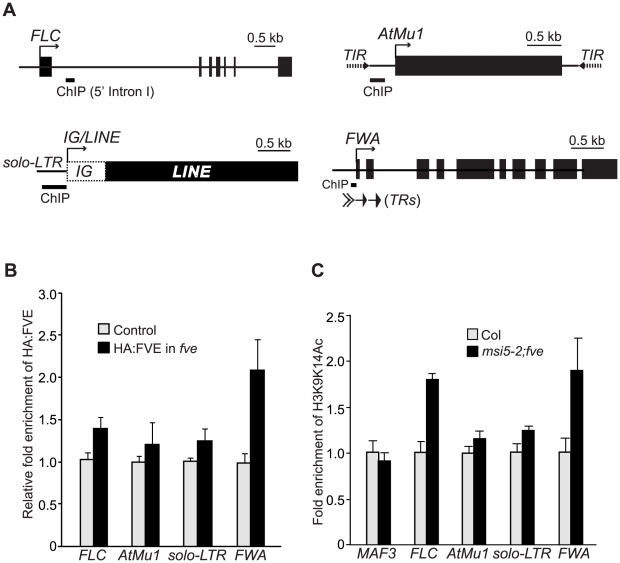
FVE Enriches at the Chromatin of Target Loci and Is Required for Histone Deacetylation. (A) Schematic structures of genomic *FLC*, *FWA*, *AtMu1* and *IG/LINE*. Solid arrows indicate the transcriptional start sites. Solid bars indicate the regions examined by ChIP. (B) HA-FVE enrichment at *FLC*, *FWA*, *AtMu1* and *solo-LTR*. Immunoprecipitated genomic DNA was measured by real-time quantitative PCR, and subsequently normalized to the constitutively expressed *TUB2* or *ACTIN2* (*ACT2*). The fold enrichments of HA-FVE protein in the *HA-FVE* line over the control line (Col) are shown. Error bars indicate SD of four biological repeats. (C) Analysis of acetylated H3 (at K9 and K14) in Col and *msi5-2;fve* seedlings. Immunoprecipitated genomic DNA was quantified and normalized to *TUB2* or *ACT2*. The fold enrichments of acetylated H3 in *msi5-2;fve* relative to Col are shown. Error bars indicate SD of two biological repeats (each quantified in triplicate). Note that *MAF3*, an *FLC* homolog, was included as a negative control to show that H3 is not hyperacetylated in a non-target gene upon loss of *FVE* and *MSI5* function.

To investigate whether FVE could bind to the chromatin of genes that exhibit altered expression in *fve* mutants, we performed chromatin immunoprecipitation (ChIP) experiments using the *HA-FVE* line. Using anti-HA antibodies, we immunoprecipitated DNA fragments from HA-FVE-expressing seedlings (wild-type Col was used as a negative control), and quantified DNA fragments from *FLC* [the 5′ Intron I region that is essential for *FLC* silencing; see [26]], *AtMu1* (the promoter region immediately downstream the 5′ *TIR*), *solo-LTR* and *FWA* (a region in the silencing tandem repeats) (Figure 9A). Compared with the control, the abundances of HA-FVE protein associated with *FLC*, *solo-LTR* and *AtMu1* chromatin increased in the *HA-FVE* line (Figure 9B). In addition, FVE was strongly enriched in *FWA* (Figure 9B). Of note, the moderate enrichment of HA-FVE at *AtMu1* and *solo-LTR* is likely due to that in the ChIP experiments, anti-HA may not bind effectively to the single HA epitope tag fused to FVE at these *TE*-containing loci. Taken together, these data suggest that FVE directly interacts with *FLC* and the three RdDM loci.

FVE or MSI5 forms an HDAC complex with HDA6 and may mediate histone deacetylation for transcriptional silencing. Hence, we examined H3K9 and K14 acetylation state in *FVE* and *MSI5* targets in WT and *msi5-2;fve* seedlings. H3K9K14 acetylation levels were moderately increased in the *AtMu1* promoter region and *solo-LTR*, and were strongly elevated in the 5′ Intron I of *FLC* and *FWA* tandem repeats upon combined loss of *FVE* and *MSI5* function (Figure 9C), consistent with FVE enrichments at these regions. Together, these data suggest that *FVE* and *MSI5* are required for histone deacetylation at *FLC* and the three RdDM target loci. Given the association of HDA6 with FVE and MSI5, these two proteins may act as part of the HDA6-containing HDAC complexes to mediate deacetylation of target loci and silence their expression.

## Discussion

In this study, we show that *MSI5* acts redundantly with *FVE* to silence various loci targeted by siRNAs or asRNAs, including *FLC* and RdDM target loci. Both FVE and MSI5 form HDAC complexes with HDA6 to mediate histone deacetylation in their target loci. In addition, these two genes function in de novo CHH methylation and maintenance of symmetric cytosine methylation at the endogenous RdDM target loci, and are also required for the establishment of cytosine methylation of the previously unmethylated sequences. Our findings suggest that FVE or MSI5 acts in the context of HDA6-containing co-repressor like complexes to mediate chromatin silencing of developmental genes and RdDM loci of transposable and repetitive elements.

### Role for FVE/MSI5-HDA6 in Cytosine Methylation

*FVE* and *MSI5* are required for cytosine methylation at three representative RdDM target loci. These two proteins may mediate cytosine methylation partly via FVE/MSI5-HDA6 complex-catalyzed histone deacetylation. HDA6-catalyzed histone deacetylation is known to be required for cytosine methylation in transgenes and endogenous *rRNA* genes. It has been shown that silencing of a transgene promoter targeted by RdDM requires *HDA6* [13]. The absence of HDA6 activity causes a substantial decrease in symmetric CG and CHG methylation and a moderate decrease in CHH methylation in the promoter region, leading to reactivation of the silenced transgene [13]. Recent studies have shown that *HDA6* exhibits a complex interrelationship with cytosine methylation in silenced *rRNA* genes: loss of HDA6 activity leads to a decrease in symmetric CG and CHG methylation and de-repression of intergenic transcription resulting in overproduction of siRNAs and consequent increase in CHH methylation [11]. Thus, *HDA6* is not essentially required for CHH methylation at *rRNA* genes; however, this does not exclude that *HDA6* is still partly involved in this methylation at loci other than *rRNAs*. A very recent study has revealed that *HDA6* is required for both CHH and CHG methylation at a few loci, and partly for CG methylation in some of these loci in *Arabidopsis* [51].

We have found histone hyperacetylation, loss of cytosine methylation and transcriptional reactivation at the RdDM target loci *FWA*, *AtMu1* and *solo-LTR* (*IG/LINE*) upon combined loss of *MSI5* and *FVE* function. This raises the possibility of that the loss of cytosine methylation might result from transcriptional activities at these loci. However, the reasons below argue for that the loss of DNA methylation at least partly causes re-activation of these silent loci. First, both *TIR*s and *solo-LTR* are non-transcribed regions. Second, the examined regions with a loss of cytosine methylation including *TR*s in *FWA*, *TIR*s in *AtMu1* and *solo-LTR* in *IG/LINE* have been shown to generate siRNAs that trigger DNA methylation at these regions leading to transcriptional silencing [40], [42], [44], [45].

In this study, we have revealed that *MSI5* and *FVE* act to silence various loci targeted by siRNAs or asRNAs. This raises the possibility of that these genes could be involved in the production of siRNAs and asRNAs for transcriptional silencing. Recent studies show that loss of *FVE* function does not affect the production of neither asRNAs from the *FLC* locus nor the siRNAs from *AtMu1*, *solo-LTR* and *AtSN1* [30], [38]. We have measured the levels of Pol V-dependent silencing scaffold RNAs from *solo-LTR* and *AtSN1* [5], [45], in mutant seedlings carrying a knockout allele of *FVE* and/or *MSI5*, and observed that the loss of *FVE* and/or *MSI5* function does not affect the production of these RNAs (Figure S4). It has also been shown that *HDA6* is not involved in siRNA production from the promoter region of a silencing-reporter transgene [13]. Based on these findings, we infer that FVE/MSI5-HDA6 complexes act downstream of or in parallel to siRNA/asRNA production for transcriptional silencing of *FLC* and RdDM loci. De novo CHH methylation and maintenance of symmetric CHG methylation at the endogenous RdDM loci are catalyzed by the DNA methyltransferases DRM2 and CMT3, respectively, and MSI5/FVE-HDA6 complex-mediated histone deacetylation is expected to facilitate these cytosine methylations. In animals only the CG dinucleotide is methylated [1], hence, the mammalian homologs of *FVE* and *MSI5*, *RbAp46/48*, certainly do not play any roles in CHG and CHH methylation. Our findings on the roles for *FVE* and *MSI5* in cytosine methylation reveal a functional divergence of the plant RbAp46/48 relatives from animal counterparts.

In *Arabidopsis*, the RdDM pathway controls the establishment of cytosine methylation in the previously unmethylated sequences including CG, CHG and CHH contexts. DRM2 functions as part of the AGO4-containing RdDM effector complex to methylate cytosines in all sequence contexts [52]. Previously, it has been shown that de novo cytosine methylation of the *FWA* transgene newly introduced into *Arabidopsis* genome requires the entire RdDM-pathway components including RDR2, DCL3, AGO4, Pol IV and DRM2 [47]. In this study, we have found that *FVE* and *MSI5* are also required for the establishment of cytosine methylation at the incoming *FWA*. It is likely that the MSI5/FVE-HDA6 complex-mediated histone deacetylation contributes to the establishment of a repressive chromatin environment that promotes DRM2 to catalyze cytosine methylation at the previously unmethylated sequences.

### FVE/MSI5-HDA6 Co-Repressor–Like Complexes

**Figure 10:**
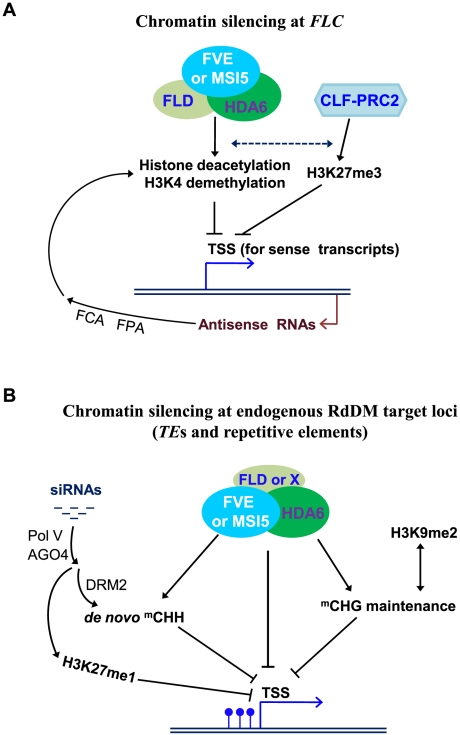
Models for Roles of *MSI5* and *FVE* in Chromatin Silencing. (A) Chromatin silencing at the development-regulatory gene *FLC*. The targeted 3′ processing of *FLC* antisense transcripts (promoted by FCA and FPA) produces RNA triggers that lead to the recruitment of repressive histone-modification activities on *FLC* chromatin. It is very likely that the RNA molecules may trigger the recruitment of FLD-FVE/MSI5-HDA6 complexes to *FLC* chromatin, resulting in repressive histone deacetylation and H3K4 demethylation. In addition, the HDA6 complexes act collaboratively with the CLF-PRC2 complex that deposits repressive H3K27me3 at *FLC*, to establish a repressive chromatin environment at the *FLC* locus leading to heterochromatin-like formation and consequent *FLC* silencing. Solid black lines with arrows indicate promotion; broken lines with arrows indicate possible promotion, and lines with bars for repression. (B) Chromatin silencing at the endogenous RdDM target loci of transposable and repetitive elements. MSI5/FVE-containing complexes mediate histone deacetylation and possibly, H3K4 demethylation, on one hand, directly represses target locus expression, and on the other hand, together with H3K9 dimethylation and/or H3K27 monomethylation, establish a repressive chromatin environment that promotes cytosine methylation (mainly CHG and CHH methylation), which may reinforce the repressive histone modifications. Together, these modifications lead to silent heterochromatin formation and consequent transcriptional silencing. FLD may act as part of the FVE/MSI5-HDA6 complexes to silence some RdDM loci. Blue lollipops indicate methyl cytosines, and ‘X’ indicates an FLD homolog (eg. LDL1) or unidentified complex component.

The mammalian FVE and MSI5 homologs, RbAp46/48, are subunits of multiple chromatin-modifying complexes such as hHAT1 (involved in gene activation), PRC2 and HDAC co-repressor complexes [16]. So far, *FVE* and *MSI5* have been found to be involved only in transcriptional silencing. Previous studies have shown that loss of *FVE* function causes reduced H3K27me3 and histone hyperacetylation at the *FLC* locus, indicating that *FVE* might act in the context of a PRC2-like complex and/or an HDAC co-repressor-like complex for *FLC* silencing under normal growth conditions [24], [26], [27]. A very recent study suggests that FVE may be part of a CLF-containing PRC2-like complex to deposit H3K27me3 in *FLC* and silence its expression [53]; however, the findings described below argue against this notion. Firstly, a gain-of-function *clf* allele *clf-59* [27], suppresses *FLC* expression in the *msi5-2;fve* double mutant, resulting in early flowering (Figure S5A); hence, *CLF* functions independently of *FVE* and *MSI5* to regulate *FLC* expression. Secondly, to genetically test whether FVE could be part of a CLF-PRC2 complex, we created a *clf;fve* double mutant in which both *clf* (*clf-29*) and *fve* (*fve-4*) are null loss-of-function alleles [19], [24], examined *FLC* de-repression in *clf*, *fve* and *clf;fve* seedlings, and found that *CLF* and *FVE* act synergistically to silence *FLC* expression (Figure S5B), suggesting that FVE may not be part of the CLF complex. Thirdly, in this study we found that *FVE* and *MSI5* silence RdDM target loci, which typically lack of H3K27me3 deposited by PRC2 complexes [54]. Lastly, we carried out co-IP experiments to determine whether FVE and CLF could be in a complex using F_1_ seedlings expressing a fully-functional GFP-CLF [55] and the HA-FVE fusion, but did not detected an association of CLF with FVE in seedlings (Figure S6). Together, these findings suggest that FVE and MSI5, unlike RbAp46/48, may not act as part of PRC2-like complexes to silence target-locus expression. At the *FLC* locus, both FVE/MSI5-HDA6 and CLF-PRC2 complexes directly repress its expression [56], and may act in concert to establish a repressive chromatin environment at *FLC* for its transcriptional silencing (see Figure 10 as described next).

In mammals, RbAp46/48 is an integral subunit of Class I HDAC co-repressor complexes [57], [58], and several of these complexes such as the BRAF-HDAC complex contain the H3K4 demethylase Lysine-Specific Demethylase 1 (LSD1) [59], a mammalian homolog of the *Arabidopsis* FLD [60]. HDA6, like the Class I HDACs, is an RPD3 (for Reduced Potassium Deficiency 3)-type histone deacetylase [13]. A recent study has revealed that HDA6 forms a complex with FLD to repress *FLC* expression and promote flowering [61]. In this study, we have found that HDA6 also forms a complex with FVE or MSI5. Together, these findings led us to infer that HDA6 and FLD form an HDAC co-repressor like complex with FVE or MSI5. Consistent with this, in a co-IP experiment using a line expressing a fully functional FLD-myc [62] and the MSI5-YFP-HA fusion, we have confirmed that indeed FLD is in a complex with MSI5 in *Arabidopsis* seedlings (Figure S7). Furthermore, like *MSI5*, *FVE* and *HDA6*, *FLD* is also required for the silencing of *FLC*, *FLC* homologs and the RdDM target loci including *AtMu1*, *AtSN1* and *IG/LINE* [38], [61]. Taken together, these *Arabidopsis* homologs of the mammalian Class I HDAC co-repressor complex components may form HDAC co-repressor like complexes to silence developmental genes and *TE*s.

The loss of *HDA6* function appears to cause a greater reactivation of *AtMu1* than that upon the combined loss of *FVE* and *MSI5* function, indicating that *HDA6* silences this locus partly independent of *MSI5* and *FVE*. One explanation is that *MSI1*, *MSI2*, and/or *MSI3* may also participate in *HDA6*-mediated silencing. *HDA6* plays multiple roles in *Arabidopsis* development. In addition to the acceleration of floral transition, *HDA6* is also involved in plant senescence and acts redundantly with its homolog *HDA19* to repress embryonic traits in vegetative growth [49], [63], [64], in which *FVE*/*MSI5* appears not to be involved (data not shown). These observations indicate that *HDA6* may act partially independent of *FVE* and *MSI5* to silence developmental genes in *Arabidopsis*.

### Models for the Functions of *MSI5* and *FVE* in Chromatin Silencing

*MSI5* acts redundantly with *FVE* to silence the developmental gene *FLC* and RdDM loci targeted by the silencing triggers asRNAs or siRNAs, respectively. Transcriptional silencing of these loci requires repressive chromatin modifications including histone deacetylation, H3K4 demethylation, H3K9 methylation, and/or H3K27 methylation.

At the *FLC* locus, the transcriptional silencing is triggered by *FLC* antisense transcripts and/or aberrant RNA molecules derived from the 3′ processing of asRNAs [30]. In the chromatin silencing at *FLC*, the targeted 3′ processing of *FLC* antisense transcripts produces RNA triggers that lead to the recruitment of repressive histone-modification activities on *FLC* chromatin. As noted above, FLD and HDA6 form a co-repressor like complex with FVE or MSI5. Consistent with this, *FLD* is required for both H3K4 demethylation and histone deacetylation on *FLC* chromatin [26], [32], [43]. Moreover, the loss-of-function *fld* and *fve* mutations act largely non-additively to delay the floral transition (caused by *FLC* de-repression) [38]. As illustrated in Figure 10A, it is very likely that the RNA molecules may trigger the recruitment of FLD-FVE/MSI5-HDA6 complexes to *FLC* chromatin, resulting in repressive histone deacetylation and H3K4 demethylation. In addition, the HDA6 complexes are expected to act collaboratively with the CLF-PRC2 complex that deposits repressive H3K27me3 at *FLC*. Together, these histone modifiers establish a repressive chromatin environment at the *FLC* locus leading to heterochromatin-like formation and consequent *FLC* silencing (Figure 10A).

At the RdDM target loci of transposable and repetitive elements, both cytosine methylation and repressive chromatin modifications such as histone deacetylation, contribute to transcriptional silencing. For instance, *FWA* silencing is typically caused by symmetric CG methylation [40], [41]. We have found that loss of *FVE* and *MSI5* function leads to histone H3 hyperacetylation at the endogenous *FWA* and ectopic *FWA* activation in sporocytes, but only a slight reduction in CG methylation. This suggests that MSI5/FVE-HDA6-mediated histone deacetylation plays a direct role in *FWA* silencing, in addition to promoting CHG and CHH methylation at this locus. In the chromatin silencing at the RdDM target loci (Figure 10B), MSI5/FVE-containing complexes mediate histone deacetylation and possibly, H3K4 demethylation, on one hand, directly represses target locus expression, and on the other hand, together with H3K9 dimethylation and/or H3K27 monomethylation, establish a repressive chromatin environment that promotes cytosine methylation (mainly CHG and CHH methylation), which may reinforce the repressive histone modifications. Together, these modifications lead to silent heterochromatin formation and consequent transcriptional silencing. FLD, the putative H3K4 demethylase, may act as part of the FVE/MSI5-HDA6 complexes to silence some of the RdDM loci such as *AtMu1*, *IG/LINE* and *AtSN1* because *FLD* has been shown to be required for silencing of these loci. Previously we have observed that *FLD* appears not to be required for *FWA* cytosine methylation and silencing, but two *FLD* homologs known as *LDL1* and *LDL2* mediate *FWA* silencing [43]. It is likely that HDA6 and FLD may form a co-repressor like complex with FVE or MSI5 to silence certain RdDM target loci, whereas at some other loci, HDA6 and FVE/MSI5 may form a complex with other components for transcriptional silencing.

## Materials and Methods

### Plant Materials and Growth Conditions

*Arabidopsis thaliana fve-4* [24], *flc-3* [65], *hda6/axe1-5* [49], *clf-29* [19] and *clf-59* [27] were described previously. The *msi5-1* (Salk_004926) and *msi5-2* (Salk_116714) alleles were isolated from the SALK collection [66]. Plants were grown under cool white fluorescent lights in long days (16-hr light/8-hr dark) or short days (8-hr light/16-hr dark).

### Bimolecular Fluorescence Complementation Assay

The full-length coding sequences for HDA6, FVE and MSI5 were translationally fused with either an N-terminal EYFP fragment in the *pSAT1A-nEYFP-N1/pSAT1-nEYFP-C1* vectors and/or a C-terminal EYFP fragment in the *pSAT1A-cEYFP-N1/pSAT1-cEYFP-C1-B* vectors (www.bio.purdue.edu/people/faculty/gelvin/nsf/index.htm). Using the Helium biolistic gene transformation system (Bio-Rad), onion epidermal cells were transiently co-transformed by appropriate plasmid pairs as indicated in Figure 7. EYFP fluorescence in the onion cells was observed and imaged using a Zeiss LSM 5 EXCITER upright laser scanning confocal microscopy (Zeiss) within 24–48 hrs after bombardment.

### Plasmid Constructions

To create *HA-FVE* fusion, the full-length *FVE* coding sequence (1.5 kb) was first cloned into the entry vector *pENTR4* (Invitrogen), and subsequently, the *FVE* fragment was inserted downstream of the *35S* promoter and the single *HA* epitope in the *pEarlyGate 201* vector [67] via gateway technology (Invitrogen), resulting in the *p35S-HA-FVE* plasmid. For *MSI5-YFP-HA* construction, the full-length *MSI5* coding sequence (1.5 kb) was inserted downstream of the *35S* promoter, but upstream of *YFP* followed by the single *HA* epitope in the *pEarlyGate 101* vector [67], resulting in the *p35S-MSI5-YFP-HA* plasmid. To construct *GST-HDA6*, the full-length *FVE* coding sequence was cloned into downstream of *GST* in the protein expression vector *pGEX-4T-1*. To construct *FVE-GUS*, a 4,073-bp *FVE* genomic fragment (from −1,886 to +2,187; A of the start codon as +1) including a 1.9-kb native promoter plus a 2.2-kb genomic coding region was inserted upstream of the *GUS* reporter gene in the *pMDC162* vector [68]; the genomic coding sequence was in frame with *GUS*. For *MSI5-GUS* construction, we inserted a 2,145-bp *MSI5* genomic fragment (from −438 to +1,707) into upstream of the *GUS* reporter gene in *pMDC162*; the genomic coding sequence of *MSI5* was in frame with *GUS*. For the construction of a binary plasmid harboring a wild-type copy of *MSI5*, a 5.1-kb genomic fragment including the 5′ promoter (1.6 kb), genomic coding sequence (3.2 kb) and 3′ end (0.3 kb), was cloned into *pBGW* [69]. To clone the gain-of-function *clf-59* allele with a single point mutation [27], a 6.5-kb genomic fragment of *clf-59* consisting of a 1.3-kb 5′ promoter, 4.5-kb genomic coding sequence and 0.7-kb 3′ end, was amplified from a Ws background and cloned into the binary vector *pHGW* [69].

### Real-Time Quantitative RT-PCR

Total RNAs were extracted from aerial parts of 10-d-old seedlings grown in long days as described previously [43]. The total RNAs were subsequently treated with ‘TURBO DNA-Free’ (Ambion) to remove residual genomic DNA. After reverse transcription, the real-time quantitative PCR was carried out on an ABI Prism 7900HT sequence detection system as previously described [43]. Primers used to amplify the cDNAs of *FLC*, *FLM*, *MAF2-5*, *IG/LINE*, and *TUB2* (At_5g62690) have been previously described [38], [70]. The primer pairs used for *MSI5*, *FVE*, *FWA*, *AtMu1*, *AtSN1* and *Ta3* amplification are specified in Table S1. Each sample was quantified in triplicate and normalized to the endogenous control *TUB2*. Bars indicate standard deviations of triplicate measurements.

### Bisulfite Genomic Sequencing

DNA was extracted from 10-d-old seedlings grown in long days, and subsequently, approximately 0.2-µg genomic DNA from each genotype was treated with bisulfite using the EpiTect Bisulfite kit (Qiagen) according to the manufacturer's instruction. The bottom strands of the endogenous *FWA* (tandem-repeat region), *solo-LTR* and *AtMu1* (the 3′ terminal-inverted-repeat region) were amplified by PCR, and cloned into the *T-Easy* vector (Promega). The primers used for the endogenous *FWA* amplification has been described previously [43], and the primer pairs for *solo-LTR* and *AtMu1* amplification are specified in Table S1. Analysis of cytosine methylaion of the *FWA* transgene in T_1_ transformants of *flc* and *flc;msi5;fve* was performed as described previously [40], [43].

### Protein Pull-Down Assay

Total proteins were extracted from 10-d old seedlings. Briefly, 0.5-g seedlings were ground in liquid nitrogen and homogenized in 1.0-ml extraction buffer (50 mM Tris-HCl pH 7.4, 100 mM NaCl, 10% glycerol, 0.1% NP-40, 1.0 mM PMSF) supplemented with 1× Roche protease inhibitor (without EDTA). Subsequently, the GST-HDA6 or GST proteins affinity-purified from the *E.coli* strain *BL21* (*DE3*) together with the glutathione-linked resins (Sigma) were added into 1.0-ml protein extracts and incubated for 4 hrs at 4°C. The protein pull-downs were analyzed by immunoblotting using anti-HA (Roche, Cat#: 12-013-819-001) or anti-FLAG (Sigma, Cat#: A8592).

### Co-Immunoprecipitation

Immunoprecipitation experiments were performed as described previously [71]. Briefly, 0.4-g seedlings were harvested and ground in liquid nitrogen, and subsequently, total proteins were extracted and immunoprecipitated with anti-FLAG M2 affinity gel (Sigma, Cat#: A2220). Proteins in the immunoprecipitates were detected by western blotting with anti-FLAG (Sigma, Cat#: A8592) or anti-HA (Roche, Cat#: 12-013-819-001).

### ChIP-Quantitative PCR

ChIP experiments were performed with 10-d-old seedlings largely as previously described [72], [73]. Briefly, nucleus fraction was isolated, and subsequently, immunoprecipitations were carried out using the polyclonal anti-acetylated histone H3 (Lys 9 and Lys 14) (Millipore, Cat#: 06-599B) or anti-HA (Sigma, Cat#: H6908). Quantitative measurements of genomic fragments of *FLC*, *AtMu1*, *solo-LTR*, *MAF3* and *TUB2* (as the internal normalization control) were performed using SYBR Green PCR master mix (Applied Biosystems). Quantitative measurements of *FWA* genomic regions and *ACTIN2* (At_3g18780; served as the internal normalization control for *FWA* enrichment) were performed on an ABI Prism 7900HT sequence detection system using TaqMan MGB probes (FAM dye–labeled) as described previously [43]. Each ChIP sample was quantified in triplicate. The primers used to amplify *FLC* and *TUB2* were described previously [43], and the primer pairs for *AtMu1*, *solo-LTR* and *MAF3* amplification are specified in Table S1. Rationale for calculation of the fold enrichment of HA-FVE in the *HA-FVE* line over the control line (Col) is as follows: in the *HA-FVE* line a gene of interest (*eg. FLC*) was first normalized to *TUB2* or *ACTIN2*, and in the control line the gene of interest was similarly normalized; subsequently, the normalized value from the *HA-FVE* line was further normalized by the value from the control line to obtain a value of fold enrichment for the gene of interest. A similar rationale was adopted for the calculation of fold enrichment of acetylated H3 in *msi5-2;fve* over Col.

## Supporting Information

Figure S1 Alignment of *Arabidopsis* FVE (At_FVE) and MSI5 (At_MSI5) with *Homo sapiens* RbAp48 (Hs_ RbAp48). Numbers refer to amino acid residues. Identical residues among these proteins are shaded black, whereas similar residues are shaded gray.
(EPS)

Figure S2 Analysis of Cytosine Methylaion at *FWA-TR*s, *AtMu1-TIR* and *solo-LTR*. Genomic DNA was digested with the methylation-sensitive restriction endonucleases *Fnu4HI* or *AluI*, and followed by PCR. *Fnu4HI* reports CHG and CG methylation at *FWA*, whereas *AluI* reports CHG and CHH methylation at *AtMu1* and *solo-LTR*, respectively. *TUB2* and *UBQ10* served as controls to indicate approximately equal amounts of DNA were used for all reactions.
(EPS)

Figure S3 Biological Functional Analysis of the Epitope-Tagged *FVE*, *MSI5* and *HDA6*. Transgenic lines were grown in long days, and total leaf number for each line (9–15 plants per line) was scored. Error bars indicate SD. (A) Flowering times of *msi5* and the transgenic line of *p35S-MSI5-YFP-HA* in *msi5* (T_3_ homozygotes). (B) Flowering times of *fve* and the transgenic line of *p35S-FVE-FLAG* in *fve* (T_3_ homozygotes). (C) Flowering times of *hda6* and *HDA6-FLAG*-expressing lines (in the *hda6* background; T_2_ generation). Note that the line with fully-functional *p35S-HDA6-FLAG* was subsequently crossed to the *HA-FVE* line, whereas the *pHDA6-HDA6-FLAG* line was crossed to the *MSI5-YFP-HA* line (for co-IP assays). (D) Flowering times of *fve* and *p35S-HA-FVE* in *fve* (Line #1; T_2_ generation).
(EPS)

Figure S4 Analysis of Pol V-Dependent Non-coding Scaffold Transcripts at the *solo-LTR* and *AtSN1* Loci by RT-PCR. The indicated amplified regions of these scaffold transcripts have been described previously [5], [45]. siRNA boxes indicate siRNA-producing regions. *TUB2* served as an internal control.
(EPS)

Figure S5 Analysis of *clf-59* Function in the *fve;msi5* Mutant and Genetic Interaction of *clf-29* with *fve*. (A) The gain-of-function *clf-59* allele suppresses the late-flowering of *fve;msi5-2*. The *clf-59* allele was cloned from a Ws background [27] and introduced into *fve;msi5-2* via *Agrobacterium*-mediated transformation; subsequently the flowering times of T_1_ transgenic lines were scored. 11, 11 and 22 plants were scored for Col, *fve;msi5-2* and the T_1_ population, respectively. Bars indicate SD. (B) Relative *FLC* transcript levels in Col, *clf-29*, *fve* and *clf-29;fve* seedlings measured by real-time quantitative PCR. *clf-29* is a null loss-of-function allele. Relative expression to parental Col is presented, and error bars for SD.
(EPS)

Figure S6 FVE Does not Associate with CLF in Seedlings, as Revealed by Co-Immunoprecipitation Analysis. A transgenic line expressing a fully-functional *p35S-GFP-CLF* was crossed to the *p35S-HA-FVE* line, and from the resultant F_1_ seedlings total proteins were extracted. Subsequently, HA-FVE proteins were immunoprecipitated with anti-HA agarose, and the precipitates were further analyzed by western blotting with anti-GFP. GFP-CLF was not detected in the HA-FVE precipitates.
(EPS)

Figure S7 FLD Associates with MSI5 in *Arabidopsis* Seedlings. Total protein extracts from a transgenic line expressing a functional *FLD-myc* [62] (served as a control) or the *FLD-myc* line carrying *p35S-MSI5-YFP-HA*, were immunoprecipitated with anti-HA (recognizing MSI5-YFP-HA) agarose; subsequently, the precipitates were analyzed by western blotting with anti-HA and anti-myc. Note that no association of FLD-myc with YFP-HA was detected in an unrelated co-IP assay (data not shown).
(EPS)

Table S1 List of Primers Used in RT-PCR, ChIP-qPCR and Bisulphite Genomic Sequencing.
(DOC)
