# Supplementary material for: Arabidopsis Homologs of Retinoblastoma-Associated Protein 46/48 Associate with a Histone Deacetylase to Act Redundantly in Chromatin Silencing
Source: PLoS Genet. 2011 Nov 10;7(11):e1002366. doi: 10.1371/journal.pgen.1002366 (PMC3213158; doi:10.1371/journal.pgen.1002366)
Supplement: Table S1 — List of Primers Used in RT-PCR, ChIP-qPCR and Bisulphite Genomic Sequencing. (DOC) [file pgen.1002366.s008.doc]

**Table S1.** List of Primers Used in RT-PCR, ChIP-qPCR and Bisulphite Genomic Sequencing

Experiments Amplified regions Sequences

**RT-PCR** *MSI5* (full-length) Forward: AGGAAATGGAAAGCGAAGCAGC

Reverse: AAGACTTGGAGGTACATGTGAAAACATGTG

*MSI5* (qPCR) Forward: CGTCTGGCCCTCTCTCTCTTG

Reverse: TGACCAAAGTATTAGGCACACTGC

*FVE* (qPCR) Forward: CTCGTCTGGCCTTCACTCTCT

Reverse: GCAACCCTTGGCTTAACAACTTC

*FWA* Forward: AAGCCTCTCGACCCTTTCAT

Reverse: TTTGCAGACAATCCTGGACTAAT

*AtMu1* Forward: AGAGTGGATATACCAAAAACACAATGT

Reverse: CCTTCTTAGCCTTCTTTTCAATCTGAGTA

*AtSN1* Forward: GCAAGCCTAGTTTTAATTCTACGGATCA

Reverse: GGTAAATCTCTCAGATAGAGGTGCTG

*Ta3* Forward: GGCATTGAGAGACACAGGACC

Reverse: GTGGTTAATAGCAGAGGCAGGAG

**ChIP-qPCR** *AtMu1* Forward: GGAAAAACTTTATTAGGGTCGCACTC

Reverse: GAGACTCCAACCAAATTACGTTTCAGAT

*solo-LTR* Forward: TGCATTACAAAAACCTTCTGATTGTCAT

Reverse: GGATGTCATTATCCATCATTCATCTCTATCCAT

AAG

*MAF3* Forward: CTGTGTGAATAGAGCCTATGCGTTACC

Reverse: CTTGAACAGCATTGAGAATGTATCAACACG

**Bisulphite Genomic Sequencing**

*solo-LTR* Forward: AAAAATAAAAATAAAAACTAAAAARAATAAAACT

Reverse: ATTATTTTYTAAGTAATTTATTTTTTAGYTAAG

*AtMu1* Forward: CTCTTTCATTCAAATTTTAATTTTTTCCATAAC

AAAART

Reverse: AATATATATAATTTTACGTAATTAATTAATATT

AATYAAG
